# 3-D aluminum nanostructure with microhole array synthesized by femtosecond laser radiation for enhanced light extinction

**DOI:** 10.1186/1556-276X-8-477

**Published:** 2013-11-14

**Authors:** Abdul Salam Mahmood, Krishnan Venkatakrishnan, Bo Tan

**Affiliations:** 1 Department of Mechanical and Industrial Engineering, Ryerson University, 350 Victoria Street, Toronto, Ontario M5B 2K3, Canada; 2 Department of Aerospace Engineering, Ryerson University, 350 Victoria Street, Toronto, Ontario M5B 2K3, Canada

**Keywords:** Aluminum nanofibrous structure, Surface plasmon resonance, Light reflectance, Light absorption, Dwell time, Repetition rate

## Abstract

This article presents 3-D aluminum micro-nanostructures for enhanced light absorption. Periodic microhole arrays were created by firing a train of femtosecond laser pulses at megahertz pulse frequency onto the surface of an aluminum target at ambient conditions. The laser trains ablated the target surface and created microholes leading to the generation of deposited nanostructures inside and around the microholes. These micro-nanostructures showed enhanced light absorption, which is attributed to surface plasmonics induced by the generation of both nano- and microstructures. These micro-nanostructures may be promising for solar cell applications.

## Background

Plasmonics is currently one of the most fascinating and fast-moving fields of photonics [1]. A variety of approaches have been developed and examined to exploit the optical properties of metallic and dielectric nanoparticles (particularly those associated with surface plasmon polariton resonances) to improve the performance of photodetectors and photovoltaic devices [1,2]. Surface plasmon resonance is the collective oscillation of electrons [3-5]. The electrons' mode of oscillation can be controlled by the shape and size of nanoparticles which, in turn, alter the optical properties such as scattering or absorptance [4].

Since the publication of a physical review article by Bethe, titled the 'Theory of diffraction by small holes’ [6], many researchers have investigated the optical transmission properties of nanohole arrays with various metals and dielectrics [7-11].

Yu et al. proposed employing silicon-on-insulator photodetector structures to investigate the influence of nanoparticle periodicity on coupling of normally incident light with the silicon-on-insulator waveguide. An enhancement of photocurrent by a factor as large as 5 to 6 was obtained due to the local surface plasmon resonance [2]. For instance, Kelly et al. used the discrete dipole approximation (DDA) method for solving Maxwell's equations for light scattering from particles of arbitrary shape in a complex environment [12].

Maier presented a study that quantified nanostructure properties (i.e., local surface plasmon resonance energy, dephasing/lifetime, total cross section, and contribution of scattering and absorption of light) of aluminum (Al), with supported nanodisks as the model system [5].

Many suitable metals have been examined for the generation of local surface plasmon resonance (LSPR). Most of them are noble metals like gold, platinum, and silver. Aluminum is a particularly interesting material from both fundamental and application points of view. It is more abundant and cheaply available than the noble metals [5]. More importantly, it fulfills the requirement for LSPR, where large negative real parts and a small dielectric imaginary part are needed (i.e., negative dielectric permittivity *ϵ*_m_ < 0) [4,10]. Therefore, aluminum nanostructures are more likely to support LSPRs for a longer period of time with high optical cross sections, wherein the excitations can be tuned over a wide energy range. Sámson provided a detailed discussion of the basic features of the plasmon resonances of aluminum nanoparticles and the free-standing aluminum hole arrays, highlighting their differences from Au and Ag nanoparticles [1].

Traditionally, nanohole arrays are fabricated by beam lithography, evaporation, and chemical catalytic methods. This work has proposed a new approach, where an ultrafast laser is used to ablate the surface of bulk aluminum. This high-intensity laser pulse delivered at megahertz frequency simultaneously creates a periodic microhole array as well as large deposition of nanostructured aluminums.

## Methods

### Experiment

A direct diode-pumped Yb-doped fiber oscillator/amplifier (*λ* = 1,064 nm) system capable of producing variable energies of up to 18.5 W at a pulse repetition frequency between 25 kHz and 200 MHz was used to drill the periodic microhole arrays.

Samples are bulk aluminum plates of 10-mm^2^ area and 2.5-mm thicknesses. They were cleaned and electropolished by 2% HF before the ablation. A linearly polarized irradiation laser beam of 1,030-nm wavelength was focused using a concave lens of 12.5-mm focal length. The pulse frequencies were set at 4, 8, 12, and 26 MHz and dwell times at 0.1, 0.25, 0.5, and 1 ms. The entire experiment was conducted under ambient conditions. The best particle quality was obtained at 26 MHz, with minimum microsized particles and a well-formed weblike structure. Unless specified otherwise, the results presented in this article are all from 26-MHz repetition rate.

The morphology of all ablated samples was examined by scanning electron microscopy (SEM), energy-dispersive X-ray (EDX) analysis, and transmission electron microscopy (TEM). The light reflectance and absorption intensity for wavelength range of 200 to 2,200 nm was tested using a spectrophotometer.

### Observations

#### Morphology of aluminum nanostructures

**Figure 1:**
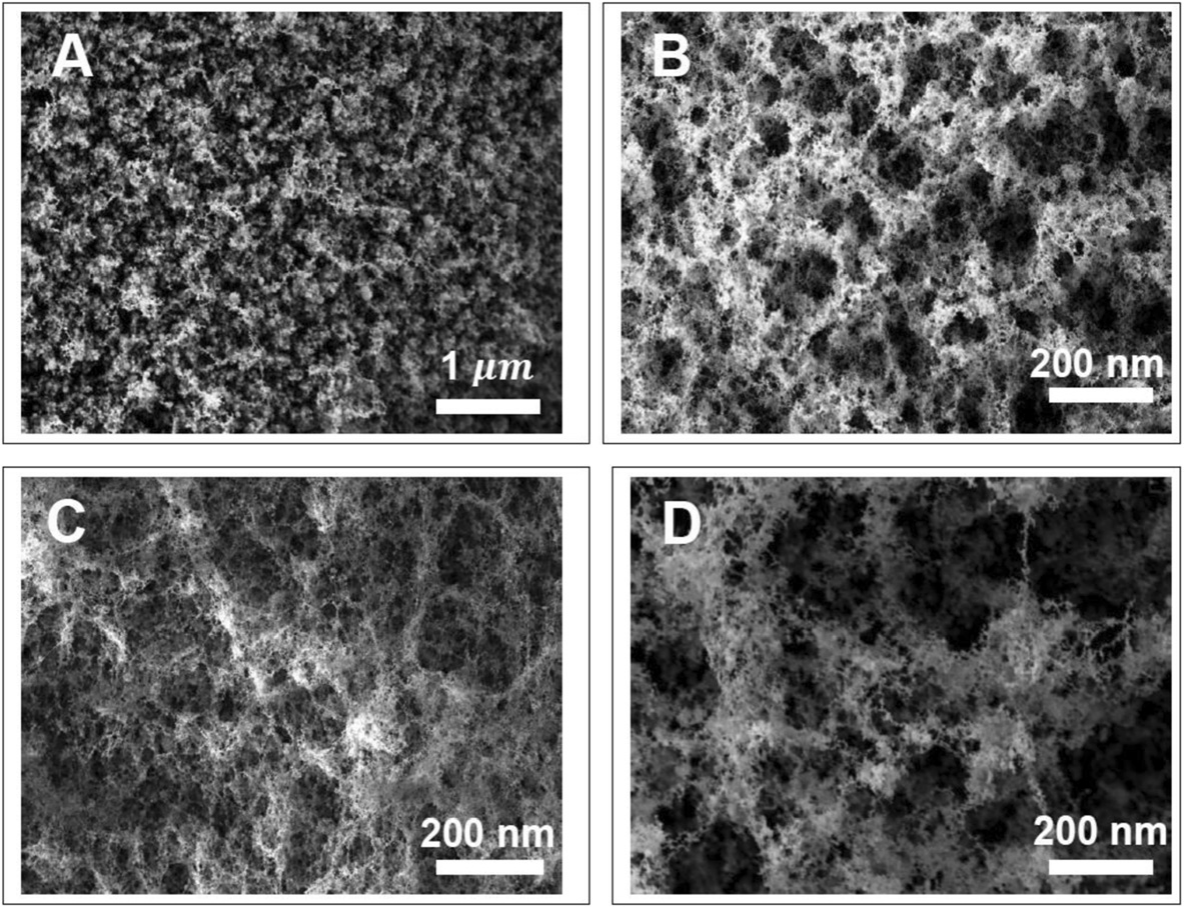
**SEM images of weblike aluminum nanofibers. (A)** 0.1, **(B)** 0.25, **(C)** 0.5, and **(D)** 1 ms of laser dwell time.

**Figure 2:**
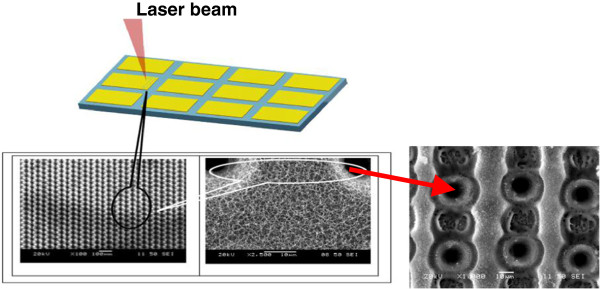
Microhole array and Al nanofiber irradiated sample.

**Figure 3:**
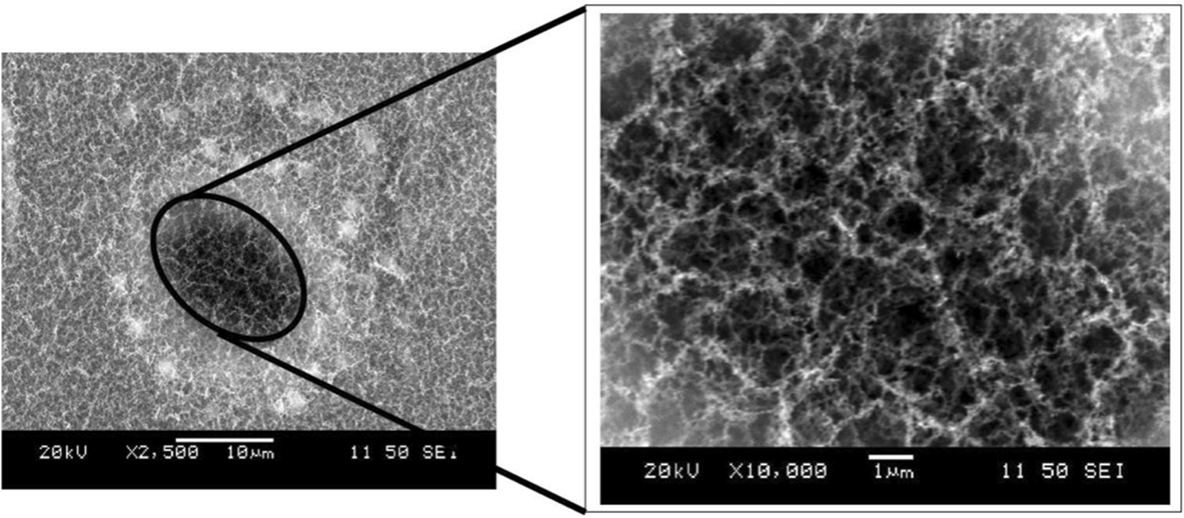
SEM images of nanofiber inside the microhole.

SEM micrographs of the irradiated surfaces around the microhole arrays are shown in Figure 1. The periodic microholes (of diameter around 10 μm) start to form with a low pulse frequency of 4 MHz (see Figure 2). Interweaved weblike fibrous nanoparticle aggregates with a certain degree of nanoporosity are also observed inside these microholes. This was consistently observed in all of the samples processed, under different conditions, during this set of experiment, as shown in Figure 3.

**Figure 4:**
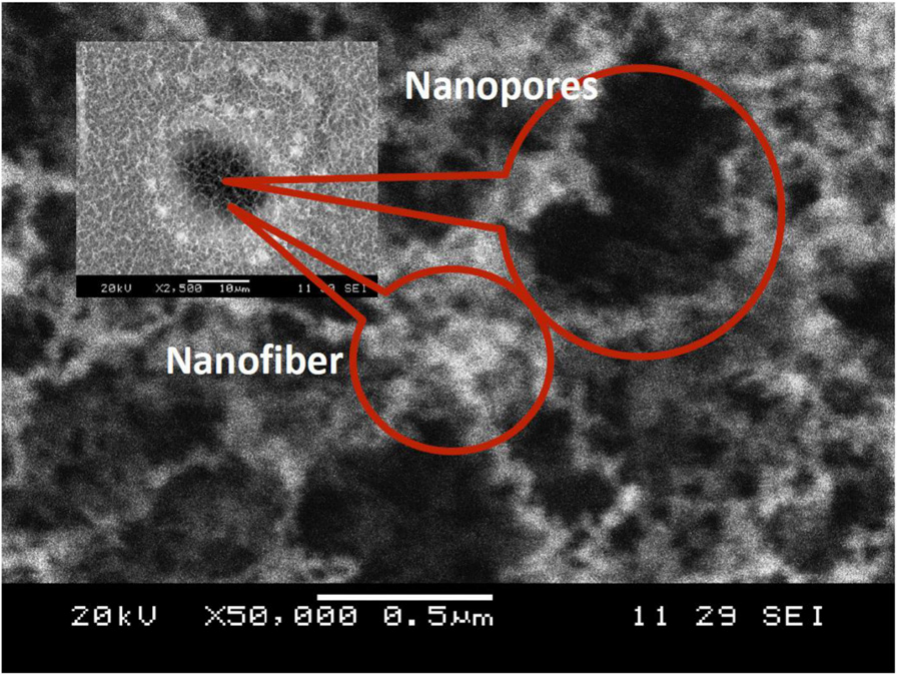
SEM images of microhole (inset) and nanofiber inside the hole.

The size of Al nanofibers in the fibrous nanoparticle aggregate structure is as small as 50 nm, as evident from the TEM analysis (see Figure 4).

**Figure 5:**
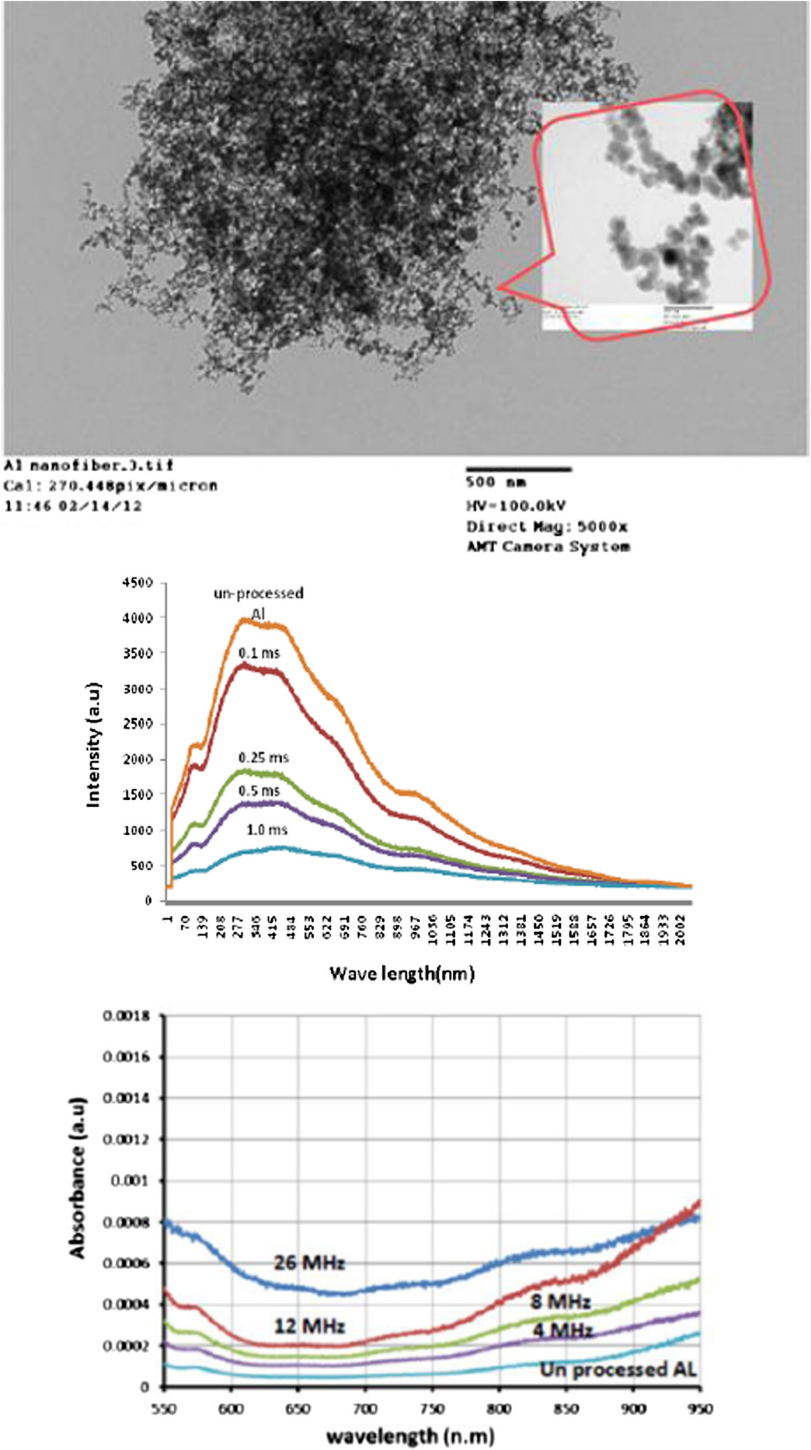
TEM images of aluminum nanoparticles.

The nucleation and generation of nanostructure features inside the microhole can be explained by the 'Raizerzelodive (RZ) theory.’ It is the most prevalent theory of dynamic condensation of expanding vapor through ultrafast laser ablation. This theory was outlined in more detail in [13]. The structures have a self-assembled weblike appearance with high dwell time, as shown in Figure 5.

**Figure 6:**
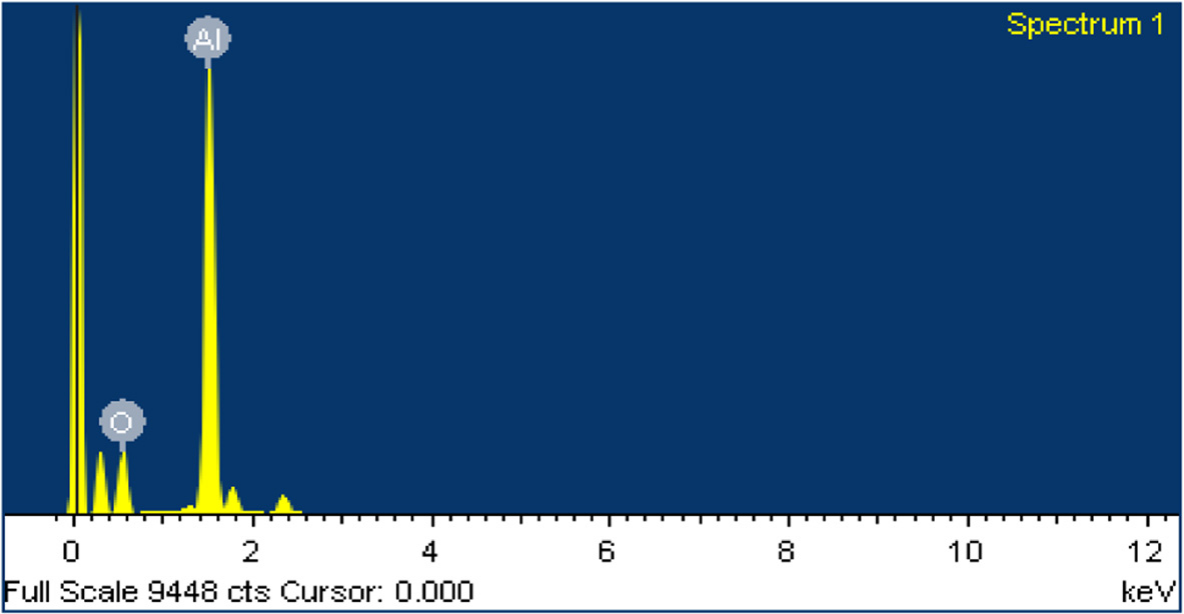
EDX spectrum of the irradiated surface showing the oxide content.

The thickness of the fibrous nanostructured layer increases as a function of the laser dwell time. Thicker depositions have a larger surface area, as illustrated in a previous work [14]. EDX revealed that the aluminum content was accompanied with oxide content (as shown in Figure 6), indicating the formation of alumina. This surface oxidation of nanostructures increases after an extended period of exposure to air. The formation of a thin 2- to 3-nm native oxide layer on an Al surface is almost instantaneous after its exposure to (humid) air [15]. The oxidation process, as well as the chemical composition and the resulting microstructure, is far more complex as a result [15,16].

#### The optical properties of aluminum nanostructures

**Figure 7:**
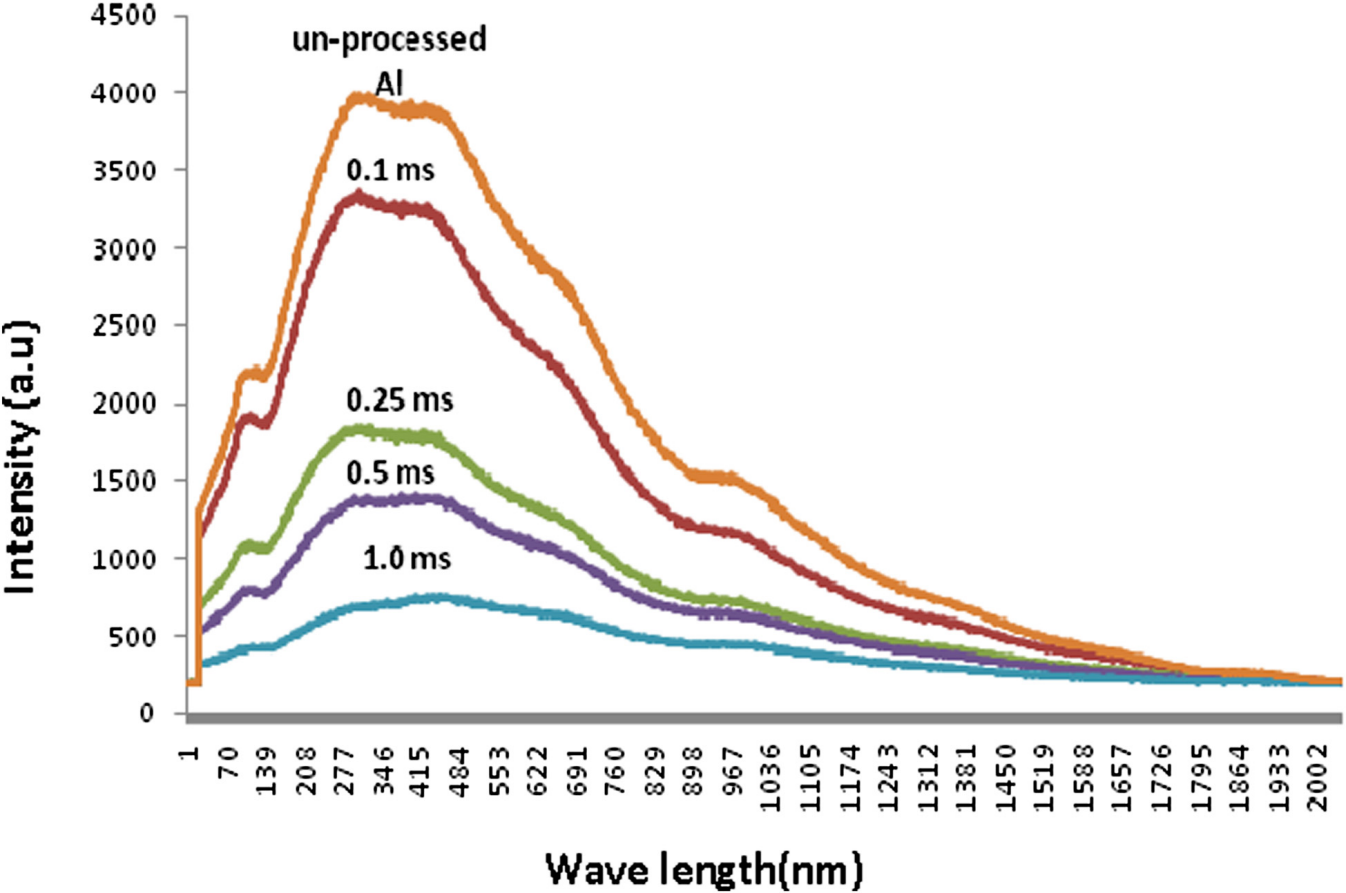
Reflection as a function of wavelength with different dwell times.

The optical properties of structured aluminum surfaces are of great interest in comparison to the properties of unstructured surfaces because the absorptance of structured aluminum changes over a broad range of visible wavelengths. The reflectance intensity characterized by the pulse frequency energy and dwell time is shown in Figure 7. It is clear that the reflectance reduces significantly as dwell time increases (therefore thicker deposition). Although not all non-reflected light is absorbed by the deposition, it is sure that the absorbance will increase when reflected light intensity reduces.

**Figure 8:**
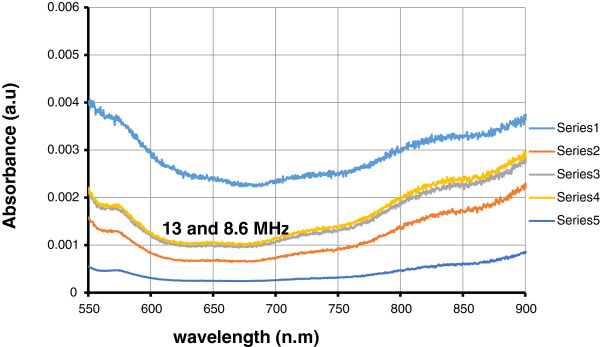
Absorption as a function of wavelength with different repetition rates.

Basically, if the holes are arranged in a two-dimensional structure within a conductive thin layer, then the transmissivity dramatically increases by over 3 orders of magnitude [17]. All irradiated samples show high absorption intensity in comparison to unprocessed samples (see Figure 8).

The strength of the enhancement could also come from a scattering center. The scattering center is the nanofiber that anchors in microholes and is close to the edges of the holes. These scattering centers decay the surface plasmon length.

The incident electromagnetic waves induce plasmon in subwavelength particles (*r* < < l, where *r* is the particle radius) and polarize the conducting electrons, resulting in collective oscillations [8]. These nanopores and nanofibrous structures that are generated inside the microholes are less than their wavelengths, as shown in Figure 4.

## Results and discussion

**Figure 9:**
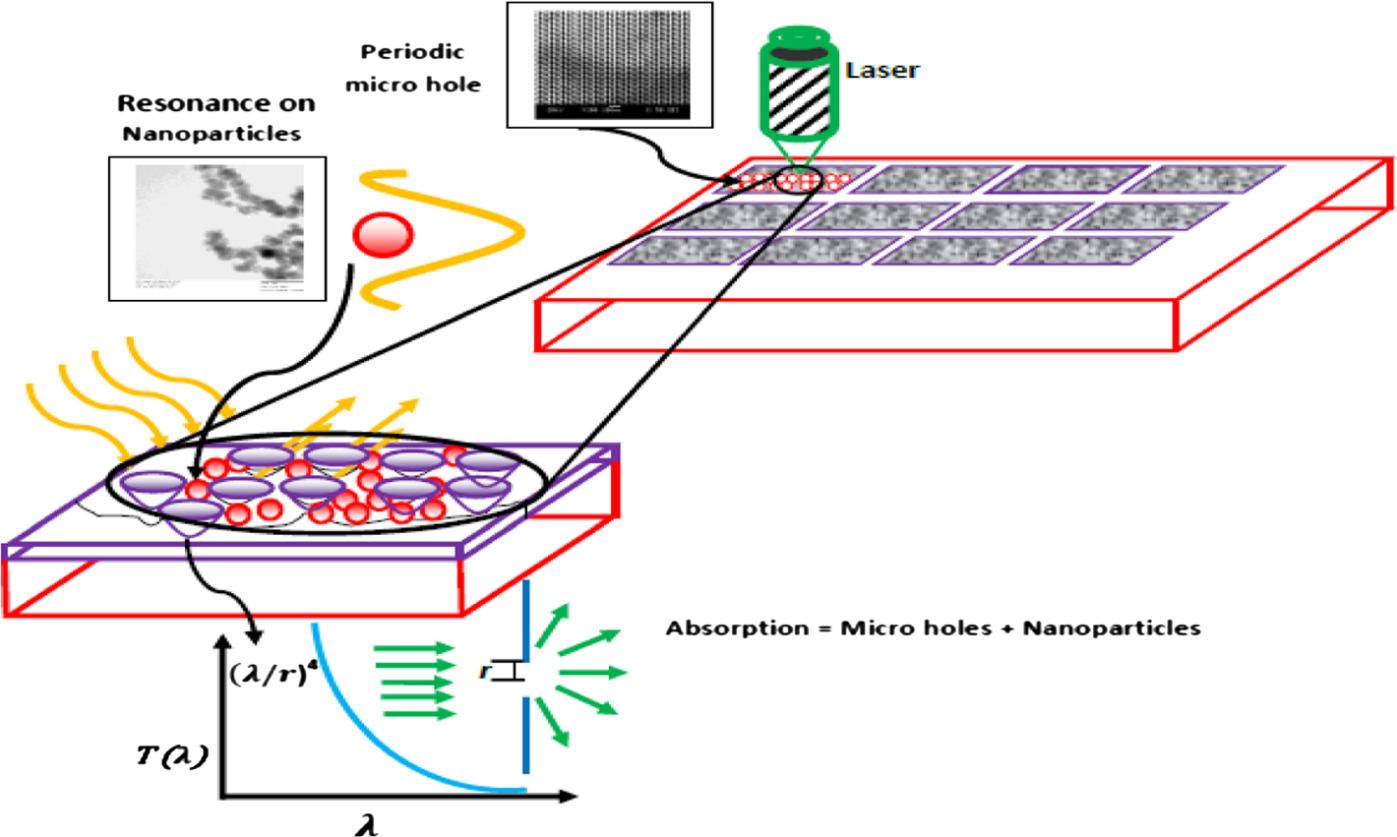
Reflection as a function of wavelength with different dwell times.

The incoming light is diffracted by the periodic hole array texture, which has closely spaced diffraction resonances where the absorption is maximized (see Figure 9) [18,19]. The maximum intensity of the optical transmission for the non-hole array depends on periodicity, as defined by the following equation:

(1) $$\;\lambda_{\max}=\frac{a_{o}}{\sqrt{i^{2}+j^{2}}}\sqrt{\frac{\epsilon_{m}\epsilon_{d}}{\epsilon_{m}+\epsilon_{d}}}\;$$

In this equation, *a*_o_ is the periodicity of holes, *ϵ*_d_ and *ϵ*_m_ are the dielectric constants of the incident medium, and *i* and *j* are the integers expressing the scattering mode indices [20,21]. Generally, plasmon represents the collective oscillations of electrons, while surface plasmon polarizations are surface electromagnetic waves that propagate in a direction parallel to the metal/dielectric (or metal/vacuum) interface. However, since the wave is on the boundary of the metal and the external medium (for example, air or any dielectric material), these oscillations are very sensitive to any change of this boundary, such as the absorption of molecules to the metal surface. The coupled light - electron oscillations on the surface of noble metal (platinum, silver, and gold) structures - is a phenomenon described by Maxwell's and Mie constitutive equations. Assuming that the particle size is very small compared to the incident wave length, the ScatLab Mie-theory software package was employed to predict the cross sections for absorption and scattering of the particle (with radius (*R*)) as follows:

(2) $$\;Q_{\mathrm{abs}}\left(\lambda \right)=\pi R^{2}4\;\left(\;\frac{2\pi n_{m}a}{\lambda }\right)\mathrm{im}\left(\frac{\epsilon -\epsilon_{n}}{\epsilon +2\epsilon_{n}}\right)$$

(3) $$Q_{\mathrm{Sca}}=\pi a^{2}\frac{8}{3}{\left(\frac{2\pi n_{m}\;a}{\lambda }\right)}^{2}{\left|\frac{\epsilon -\epsilon_{n}}{\epsilon +2\epsilon_{n}}\right|}^{2}$$

**Figure 10:**
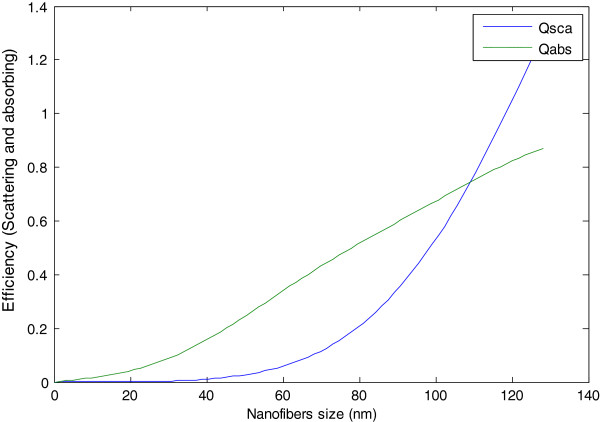
**Theoretical calculations of **$Q_{\mathrm{Sca}}$**and**$Q_{\mathrm{abs}}$**efficiencies with different particle sizes.**

In this equation, $Q_{\mathrm{abs}}$ and $Q_{\mathrm{Sca}}$ are the absorption and scattering cross sections, respectively, *λ* is the incident radiation wavelength, *a* is the scattering coefficient, *R* is the radius of the particle, and *n*_m_ is the number of molecules per unit volume at standard temperature and pressure. Consequently, the absorption cross section ($Q_{\mathrm{abs}}$) becomes the dominant process, accompanied by a large increase in the electromagnetic field amplitude for a particle size less than the incident light wavelength. According to the mathematical calculations, the maximum aluminum nanoparticle size should not be greater than 110 nm (the intersecting point of the two curves), as shown in Figure 10. The mean particle size of the aluminum nanostructure is measured to be 50 nm, which is below the critical particle size given in Figure 10, suggesting that when light passes through the nanofibrous deposition, absorption dominates over scattering.

Generating a thin homogeneous layer of aluminum nanofibrous structure on the bulk of an Al substrate will be advantageous to get an identical reflective index as it will result in a homogeneous external field that induces a dipole in the nanoparticles. Otherwise, when the nanoparticle is supported on a substrate whose refractive index is different from that of the ambient air, the field acting on the particle will no longer be homogeneous due to the image dipole field that is induced in the substrate [22].

Consequently, the laser parameters (dwell time and repletion pulse energy) will significantly affect the high reduction in reflectance intensity due to an increased nanofiber creation, due to which the Al nanofibrous structural response caused by the dipole oscillation of localized surface plasmons increases the metal excitation for incident light. This excitation enhances the local electromagnetic field near the nanofibrous layer at surface plasmon resonance and the scattering cross section for off-resonant light [23]. In addition, when nanoparticles are sufficiently close together, interactions between neighboring particles arise. Therefore, when the longer dwell time has created an intensive quantity of homogenous nanofibrous structures, the dipole created by the electric field of light will induce a surface polarization charge, which effectively acts as a restoring force for the free electrons.

## Conclusions

The effects of the aluminum nanofeatures (nanopores and nanofibers) for enhanced light absorption were studied in this article. The nanofeatures, which are generated inside and around the periodic microholes, were synthesized by femtosecond laser irradiation. The generation of the nanostructures was explained by nucleation and condensation of plasma plume grown during the irradiation process. Significant reduction in light reflection with acceptable improvement of the absorption intensity has been observed with long irradiation time (dwell time) and high repetition rate. The interaction between the small size of nanopores and the bulk quantity of nanoparticles could restore the resonance of the surface plasmons.

## Competing interests

The authors declare that they have no competing interests.

## Authors' contributions

ASM was KV and BT's Ph.D. student. ASM carried out the theoretical study and material characterization and drafted the manuscript. KV conceived of the study and carried out the experiment. BT participated in the theoretical study and conducted critical review, manuscript revision, and coordination. All authors read and approved the final manuscript.
